# Biological Functions of Interleukin-21 and Its Role in Inflammation

**DOI:** 10.1100/tsw.2007.275

**Published:** 2007-10-22

**Authors:** Martin Pelletier, Denis Girard

**Affiliations:** ^1^ Department of Pathology, Division of General Pathology, University of Verona, Italy; ^2^ INRS-Institut Armand-Frappier, Pointe-Claire, Québec, Canada

**Keywords:** cytokine, interleukin-21 (IL-21), neutrophil, monocyte, macrophage, inflammation, CD132, cell signaling

## Abstract

Interleukin-21 (IL-21), the most recently discovered CD132-dependent cytokine, is mainly produced by activated T lymphocytes, particularly the inflammatory Th_17_ subset, and is believed to be a key factor in the transition between innate and acquired immunity. In the last few years, this cytokine has been shown to modulate the functions of T, B, and NK cells, as well as cells of myeloid origin. In addition, it was demonstrated that IL-21 is a potent antitumor agent, making it a promising candidate for the development of therapeutic tools. IL-21 has also been associated with different autoimmune and inflammatory diseases, such as rheumatoid arthritis and inflammatory bowel disease. This review will summarize the biological functions of IL-21 and its potential role in inflammation.

## THE CD132-DEPENDENT CYTOKINES: AN INTRODUCTION

Adequate immune responses against invading pathogens are achieved through a network of cytokines, whose functions are to induce the development and maturation of cells from the lymphoid and myeloid lineages, and the activation of their effector functions. Cytokines are also involved in the reduction and inhibition of these responses after pathogen clearance. The CD132 (γc)-dependent cytokine family includes interleukin (IL)-2, IL-4, IL-7, IL-9, IL-15, and IL-21, and is also referred to as the IL-2 family of cytokines or the γc users. The CD132-dependent cytokines are part of the hematopoietin cytokine family and are composed of a four α-helical structure bundle. These cytokines are known to mediate their biological activities by binding to a heterodimeric receptor complex composed of the shared CD132 chain and a specific α-chain. Unlike the other members of the CD132-dependent cytokines, the IL-2 receptor (IL-2R) and the IL-15R are composed of a third shared component, CD122 (IL-2/15Rβ). CD132, CD122, CD124 (IL-4Rα), CD127 (IL-7Rα), CD129 (IL-9Rα), and IL-21Rα are all members of the cytokine receptor class I superfamily, a family composed of transmembrane proteins with four conserved cysteine residues and a Trp-Ser-X-Trp-Ser (WSXWS) motif in their extracellular portion[1,2,3]. CD25 (IL-2Rα) and IL-15Rα are not part of the hematopoietin receptor family, since they do not possess the common features of this receptor family, but both contain a short binding region called the sushi domain[4,5,6,7].

It has been proposed that cells expressing either CD25 or IL-15Rα can bind to their corresponding cytokines to form a stable receptor/cytokine complex. This complex can then be presented in "trans" to target cells that only express CD122 and CD132 low-affinity dimeric receptors[8,9,10,11]. Thus, transpresentation is a novel mechanism by which cytokine signals are transduced, and it has been proposed that this phenomenon is the dominant physiological mechanism by which IL-15 supports NK and $CD8^+$ memory T-cell homeostasis[12].

The CD132-dependent cytokines play a major role in the development, maturation, and activation of T, B, and NK cells. Briefly, IL-2 promotes the proliferation, differentiation, and survival of both NK cells and antigen-activated T lymphocytes, and increases their cytokine production and cytolytic activities[13]. In addition, IL-2 is critical in the $CD4^+CD25^+$ regulatory T-cell development[14]. IL-4 is involved in Th2-cell induction and B-cell class switching, and has inhibitory effects on the Th1 cell-mediated immune responses[15]. IL-7 plays a critical role in lymphocyte development, particularly γδ T cells, and contributes to the regulation of lymphocyte homeostasis[16]. IL-9 is particularly known as a mast cell growth factor, but is also a late-acting T-cell growth factor[17]. IL-15 plays an essential role in NK-cell development and activation, and regulates interactions between macrophages and NK cells. It is also involved in the development, homeostasis, and activation of memory T lymphocytes and T cells involved in the induction of innate immune responses, such as dendritic epidermal γδ T cells, intestinal intraepithelial lymphocytes, and NKT cells. In addition, IL-15 is known as a (potent) general inhibitor of apoptosis *in vitro* and *in vivo*[18]. Finally, IL-21, the most recently discovered CD132-dependent cytokine, is believed to be a key factor in the transition between innate and acquired immunity, and its biological functions will be described below.

The importance of CD132 in the maintenance of a general immune homeostatic state is well established; mutation(s) in the CD132 encoding gene, located on the X chromosome, result in deficiencies in both cellular and humoral immunity and cause a disease known as X-SCID (*X-linked severe combined immunodeficiency*)[19,20]. The lack of T lymphocytes and NK cells is due to defects in IL-7 and IL-15 intracellular signaling. Although the number of B lymphocytes is relatively normal, they are defective in IL-4 and IL-21 signaling, two important cytokines in B-cell physiology[21]. Similar defects in lymphocyte functions have also been reported in patients with genetic mutations in the CD132-associated tyrosine kinase Jak3[22]. These observations illustrate the essential role of the common CD132 component, and its associated signaling pathways, in the development, activation, and regulation of an efficient immune response.

## BIOLOGY OF IL-21

### Discovery and Characteristics of IL-21 and Its Receptor

#### *Discovery of IL-21 and IL-21Rα*

The specific α-chain of IL-21 receptor was discovered by two different groups. Ozaki et al. identified a novel type I cytokine receptor on the genomic sequence of a bacterial artificial chromosome clone and temporarily designated it NILR (novel interleukin receptor)[23]. Parrish-Novak et al. identified an expressed sequence tag coding for a member of the class I cytokine receptor family. This new receptor was designated IL-21R[24]. It was found that IL-21Rα possesses an amino acid sequence similar to those of CD122 and CD124. In addition, the putative extracellular region contained four conserved cysteine residues and a Trp-Ser-X-Trp-Ser (WSXWS) motif. The gene encoding IL-21Rα was also found to be physically adjacent to the IL-4Rα gene on chromosome 16[23,24]. IL-21 was identified in the conditioned medium from phorbol 12-myristate 13-acetate (PMA)/ionomycin-activated human peripheral CD3+ T cells, since it induced the proliferation of a BaF3 cell line expressing IL-21R (BaF3/IL21R). IL-21 was found to possess a four-helix-bundle cytokine domain with significant homology to IL-2, IL-4, and IL-15[24]. More recently, a novel isoform of IL-21, termed IL-$21_{iso}$, was identified in both human and mouse. This isoform was shown to be highly dependent on *de novo* protein synthesis, while the secretion efficiency was found to be much lower than that of IL-21. In spite of the differences in the C-terminal amino acid sequences between the two isoforms, IL-$21_{iso}$ showed comparable biological effects to IL-21 on immune responses[25].

## Cellular Distribution and Gene Regulation of IL-21 and IL-21Rα

IL-21 mRNA has been detected in $CD4^+$ T cells, but not in $CD8^+$ T cells, $CD19^+$ B cells, $CD14^+$ monocytes, and dendritic cells[24,26]. IL-$21_{iso}$ mRNA was also detected in $CD4^+$ T cells[25]. It has been demonstrated that murine IL-21 is preferentially expressed by $CD4^+$ T cells differentiated into a Th2 phenotype, whereas human IL-21 mRNA was detected in Th1 cells and in follicular helper T cells, a non-Th1/Th2 effector providing help for B cells[3,27,28,29]. A more detailed study showed that human IL-21 is mainly expressed by activated $CD4^+$ central and effector memory T cells, some activated Th1-polarized cells, but not Th2-polarized cells. Human IL-21 is also absent from $CD4^+Foxp3^+$ regulatory T cells ($T_{reg}$), but is expressed by $Th_{17}$-polarized cells, a distinct proinflammatory Th lineage[30]. In mouse, the proximal promoter of IL-21, through the action of NFATc2 and T-bet transcription factors, was shown to control the Th-cell-subset-specific expression of IL-21. NFATc2 binds directly the IL-21 promoter and activates its transcription in Th2 cells, while T-bet represses IL-21 transcription by inhibiting the binding of NFATc2 to the promoter in Th1 cells[31]. Interestingly, of all $CD4^+$ T cell subsets, $Th_{17}$ cells were found to be the highest producers of IL-21, suggesting a role of this cytokine in inflammatory processes[32,33]. TCR stimulation, PMA/ionomycin, IFNα/β, IL-6, and IL-12 were all shown to induce IL-21 gene expression in human T cells and a calcium mobilization alone is sufficient to mediate induction of IL-21 expression in preactivated T lymphocytes[24,30,32,33,34,35,36]. Interestingly, it was observed that IL-21 can increase its own mRNA expression, suggesting an autocrine regulation in $CD4^+$ T helper cells[33,36]. Production of IL-21 by $CD4^+$ T cells was demonstrated *in vivo* by Holm et al. who showed that IL-21 mRNA is produced by mice challenged with herpes simplex virus type 2 or lymphocytic choriomeningitis virus. IL-21 production in the spleen, which coincides with the onset of the adaptive immune response to these viral challenges, was observed in the $CD4^+$ T cell fractions[37].

Recently, it was proposed that IL-21 production may not be limited to conventional $CD4^+$ T cells. Vα14 NKT cells were shown to be the major IL-21 producers in response to *Mycobacterium bovis* bacillus Calmette Guerin (BCG)[38]. In addition, NKT cells were shown to be potent producers of IL-21 following *in vitro* stimulation via CD3 and CD28, particularly in conjunction with IL-12, or following *in vivo* stimulation with the glycolipid antigen α-galactosylceramide (α-GalCer). Interestingly, IL-21 production may not be limited to $CD4^+$ cells, since $CD4^-$ NKT cells were reported to express mRNA for this cytokine[39]. Finally, the demonstration that stromal cells from lymphoid organs can transcribe the IL-21 gene favors a role for this cytokine in regulating T-cell homeostasis[40].

IL-21Rα mRNA is expressed at low levels in resting T cells, but is rapidly induced on stimulation. For example, IL-21Rα gene expression in T cells is induced by an anti-CD3 antibody stimulation, IFNα/β and even IL-21 itself, which was shown to induce its own receptor in $CD8^+$ T cells[35,41,42]. TCR-induced IL-21Rα expression was shown to be driven by TCR-mediated augmentation of the transcription factor Sp1 protein levels and its subsequent binding to the IL-21Rα promoter[43]. IL-21Rα was further observed in B cells and its expression can be up-regulated by LPS, CD40 triggering, and TNF stimulation[5,41,44,45]. Also, IL-21Rα was detected in NK cells, where its expression was up-regulated by IL-15 and down-regulated by IFNα/β[23,35]. Other cells that were shown to express IL-21Rα include NKT cells, dendritic cells, macrophages, fibroblasts, and keratinocytes[26,41,46,47,48]. The fact that IL-21Rα was also observed in different primary leukemia and lymphoma cells[23,49,50] suggests that IL-21 may play a role in the pathophysiology of some cancers.

### *Signaling Pathways*

Although IL-21 is able to bind to IL-21Rα expressed on a CD132-deficient cell line, IL-21, in the absence of CD132, is unable to transduce any intracytoplasmic signal[51,52]. On binding to its heterodimeric receptor, IL-21, as other members of the CD132-dependent cytokines, activates the Janus kinase (Jak)/Signal Transducer and Activator of Transcription (STAT) pathway. More specifically, it has been demonstrated that Jak1 associates with IL-21Rα, while Jak3 associates with CD132[23,44,45,51,52]. Once activated, the Jaks phosphorylate tyrosine residues on the receptor subunits that serve as docking sites for STATs. STAT1 and STAT3 are predominantly activated by IL-21 and IL-$21_{iso}$[25,35,44,45,49,50,51,53,54,55,56,57,58,59,60,61]. STAT5 is also activated, but to a lesser extent[23,45,49,52,60]. Although it was reported that IL-21 can inhibit STAT4 activity by reducing STAT4 mRNA and protein levels, activation of STAT4 by IL-21 was observed in NK and T cells[27,56,62].

Activation of the Jak/STAT pathway by IL-21 has been associated with different cellular functions. IL-21–induced Jak/STAT signaling was linked to differentiation of human B cells into Ig-secreting plasma cell, while STAT3 was required for IL-21–induced IL-17 expression by $Th_{17}$-polarized cells[36,63]. The duration of IL-21–induced STAT3 activation was shown to be regulated by SOCS1 in $CD8^+$ T cells[57]. Furthermore, activation of Jak3 was shown to be essential in both the survival and mitogenic signals induced by IL-21 in B cells[52]. Finally, IL-21 was found to activate STAT DNA binding to IFNγ regulatory elements, indicating that IL-21–activated STATs are involved in the activation of the IFNγ gene and subsequent IFNγ production[35,56,60].

In addition to the Jak/STAT pathway, two other major pathways have been associated to IL-21 signaling: mitogen-activated protein kinases (MAPK) and phosphatidylinositol-3 kinase (PI-3K). It was shown that IL-21 induced the phosphorylation of Shc, an adaptor protein that initiates the MAPK pathway, and Akt, a serine/threonine kinase that mediates cellular events downstream of PI-3K[54]. Induction of ERK1/2 phosphorylation by IL-21 was observed in a promyelocytic cell line and in myeloma cells, while p38 MAPK phosphorylation in response to IL-21 was observed in intestinal epithelial cells[44,46,64]. Activation of these two pathways was also linked to cellular functions. It was demonstrated that both MAPK and PI-3K pathways contribute to IL-21–mediated proliferation while IL-21–induced IFNγ secretion by NK cells was associated to ERK1/2 activation[54,60].

## Biological Effects of IL-21

### *Lessons from Transgenic and Knockout Mice*

The generation of IL-21Rα–deficient mice (IL-$21R\alpha^{-/-}$) indicated that IL-21 is not essential for hematopoiesis, since normal lymphoid and myeloid compartments are found in these viable and fertile animals[65,66,67]. Using the hydrodynamics gene-delivery method, IL-21 was found to induce aberrant expansion of hematopoietic progenitor cells in the spleen, but was insufficient for the survival and differentiation of these cells into granulocytes or monocytes in the periphery. This further indicated that IL-21 is not required for hematopoiesis[68]. The use of knockout mice and transgenic mice has clearly showed the importance of IL-21 in the regulation and production of antibodies. IL-21 transgenic mice exhibited elevated serum IgM and IgG1, and increased surface $IgG1^+$ B cells in the spleen[69]. IL-$21R\alpha^{-/-}$ mice showed higher serum levels of IgG1 and IgG2b, and lower levels of IgE than wild-type animals but, following immunization, antigen-specific IgG1, IgG2b, and IgG3 levels decreased, whereas levels of IgE increased[66]. IL-21–deficient mice showed dramatically enhanced IgE isotype switch and clonal expansion of IgE+ cells[70]. Therefore, these results indicate that IL-21 promotes Ig isotype switching *in vivo* and is critical in the maintenance of low IgE levels under physiological conditions. Cooperation between IL-21 and IL-4 was also shown to occur in the regulation of immunoglobulin production *in vivo*, since a marked deficiency in immunoglobulin production of all classes, including IgE, was observed in mice lacking both IL-21Rα and IL-4[66].

## *Role of IL-21 in Lymphoid and Myeloid Cells*

The pleiotropic effects of IL-21 depend not only on the cell type, but also on their differentiation and activation state. It has even been suggested that IL-21 is a mediator of the transition from innate to adaptive immunity, mainly by its capability to limit the duration of activation of NK cells and to promote effector T-cell functions[65]. This section will summarize the effect of IL-21 on lymphoid and myeloid cells.

### Lymphoid Cells

#### T Cells

In general, IL-21 by itself is not mitogenic for normal T cells. Proliferative effects are rather observed when IL-21 is used as a costimulant. In this respect, increased proliferation was observed when T cells were incubated with IL-21 in the presence of an anti-CD3 antibody, IL-2, IL-7, or IL-15[24,30,42,71,72]. For resting and activated T cells, IL-21, by itself, was shown to be a survival factor acting through the PI-3K signaling pathway[40]. Proliferative effects induced by IL-21 alone were observed in leukemia cells from adult T-cell leukemia/lymphoma (ATLL), a disease caused by human T-cell leukemia virus type I (HTLV-I)[49,50].

In addition to its mitogenic and apoptotic effects, IL-21 is able to modulate naive T-cell differentiation. IL-21 has been shown to inhibit specifically the differentiation of naive Th cells into IFNγ-producing Th1 cells by repressing the expression of the T-box transcription factor eomesodermin (Eomes). This indicates that IL-21 is able to amplify a Th2 response[27,73]. The promotion of Th2-type inflammatory responses by IL-21 has further been confirmed *in vivo* using IL-21Rα–deficient mice[67,74]. A critical role has also been attributed to IL-21 in the generation of $Th_{17}$ cells. $Th_{17}$, also termed Th(IL-17) or inflammatory Th (Thi), is a newly identified subset of T cells, distinct from the Th1 and Th2 subsets, that produce IL-17, IL-17F, and IL-22. These three cytokines play a crucial role in mediating autoimmunity and inducing tissue inflammation. The differentiation of $Th_{17}$ cells requires TGFβ, IL-6, and IL-23 and depends on STAT3 and the orphan nuclear receptor RORγt[75,76,77,78]. In T cells, it was recently demonstrated that IL-21 is induced by IL-6 by means of STAT3. IL-21, in cooperation with TGFβ, is then sufficient, through STAT3-dependent up-regulation of RORγt, to induce the differentiation of $Th_{17}$ cells. Furthermore, Th cells from IL-21–, IL-21Rα–, and RORγt-deficient mice showed a lack in the generation of IL-17–producing cells, indicating that IL-21 is not only sufficient, but also necessary, in the development of $Th_{17}$ cells. In addition, IL-21 was shown to suppress Foxp3 up-regulation in naive Th cells, suggesting that IL-21, similarly to IL-6, can regulate the reciprocal development pathway of generation of $Th_{17}$ and $T_{reg}$ cells. The essential role of IL-21 in $Th_{17}$ generation was also confirmed *in vivo,* since $IL\text{-}17^+CD4^+$ cells were completely absent in lamina propria and spleen of IL-21–deficient mice, while a marked decrease in the generation of a $Th_{17}$ response was observed in IL-21Rα–deficient mice. Therefore, IL-6 and IL-21, independently and together, cooperate with TGFβ to induce $Th_{17}$ differentiation[32,33,36].

IL-21 can modulate the functions of T cells according to their activation state and their subtype. IL-21 was shown to preferentially enhance Ag-specific response of naive $CD8^+$ T cells, to up-regulate the expression of genes associated with innate immunity and Th1 response, and to enhance IFNγ production in synergy with IL-15 or IL-18[55,56,79]. Many groups also observed that IL-21, alone or in combination with IL-15, was able to increase IFNγ production and the cytotoxic activity of $CD8^+$ T

cells[42,65,80,81,82,83,84]. In addition, it has been suggested that IL-21 preserves the ability of naive $CD8^+$ T cells to respond to costimulatory ligands on future encounters with activated antigen-presenting cells (APCs) expressing the cognate Ag. In this sense, IL-21 is able to maintain CD28 expression in naive $CD8^+$ T cells that have undergone IL-15–driven homeostatic proliferation and also to increase CD28 expression in combination with IL-2[79,80,85]. In addition, IL-21 was found to provide a signal to naive $CD8^+$ T cells to differentiate, in response to Ag and costimulation, in a unique effector phenotype with high cytolytic activity, but with deficient capacity to secrete IFNγ[86]. SOCS1 was recently found to be a critical negative regulator of IL-21 signaling in $CD8^+$ T cells, by controlling their proliferation in response to synergistic stimulation by IL-15 and IL-21, suggesting that SOCS1 helps to prevent activation of potentially autoreactive naive $CD8^+$ T cells during immunoinflammatory conditions[57]. Concerning the effect of IL-21 on $CD4^+$ T cells, it was recently observed that IL-21 can render them resistant to $T_{reg}$-mediated suppression, suggesting that IL-21 could augment T-cell–activated responses in human inflammatory immune diseases[87]. IL-21 was also shown to enhance IL-17 secretion from mitogen-activated human T cells, indicating that IL-21 can contribute to inflammation and tissue remodeling[88]. Finally, it was suggested that activated γδ T cells, costimulated with IL-21, played a role in lymphoid follicles and in B-germinal center physiology. In this regard, γδ T cells moving into the germinal centers were proposed to respond to follicular helper T-cell–derived IL-21 by acquiring a lymphoid-homing phenotype and by producing CXCL10/IP-10 and CXCL13/BCA-1. Therefore, γδ T cells contribute to the molecular definition of the B zone and aid further recruitment of $CXCR5^+$ B cells, follicular helper T cells, and monocytes[89].

## B Cells

On stimulation, it was demonstrated that IL-21 inhibited the proliferation of B cells induced by IL-4 and an anti-IgM antibody (which mimics the BCR activation), while increasing the proliferation induced by an anti-CD40 antibody (which mimics the CD40-CD154 interaction between B and T cells)[24,59,69,90]. In addition, IL-21 induced the apoptosis of resting and activated B cells, even in the presence of prosurvival factors, such as IL-4, IL-15, LPS, or anti-CD40 antibody, and also apoptosis of follicular B lymphoma cell lines[45,50,90]. A similar phenomenon was observed when B-cell chronic lymphocytic leukemia (B-CLL) cells were treated with IL-21 and CpG oligonucleotides, since these cells underwent apoptosis and were able to induce apoptosis of untreated bystander B-CLL cells through granzyme B production[91]. IL-21 was shown to induce Bcl6 expression, a transcriptional repressor factor essential in the formation of B-germinal centers, but whose expression could be implicated in the regulation of apoptosis[58,69,92]. In addition, the gene coding for IL-21Rα was associated with Bcl6 translocations and enhanced Bcl6 expression in the diffuse large B-cell lymphoma[93]. IL-21 also down-regulated expression of the antiapoptotic proteins Bcl-2 and Bcl-$X_L$, and up-regulated the expression of the adaptor protein hematopoietic Src homology 2 (HSH2) and the proapoptotic proteins Bim and Bax, which all results in B-cell apoptosis[41,50,90,94]. BCG-mediated Bε cell apoptosis was showed to be due to the augmented formation of proapoptotic protein Bmf and antiapoptotic protein Bcl-2 complexes generated by IL-21/IL-21R signaling in Bε cells[38]. Jin et al. proposed a model in which IL-21 represents an important checkpoint for a productive B-cell response. IL-21 promotes self-tolerance by inducing apoptosis of self-reactive B cells or B cells solely stimulated through Toll-like receptors (TLRs), while promoting proliferation, differentiation, and immunoglobulin production of B cells receiving costimulation signals[41]. Antiapoptotic and/or growth-promoting effects of IL-21 were also observed in human multiple myeloma cells, a neoplasia of terminally differentiated B cells, and Burkitt lymphoma cell lines *in vitro*[44,50]. Thus, these results indicate that IL-21, or its specific receptor, could represent a target for future therapy in B-cell–related cancers.

It was observed that IL-21 induced the differentiation of B cells into plasma cells and increased IgG production through Jak/STAT signaling, c-Myc regulation, and up-regulated expression of both activation-induced cytidine deaminase (AID) and B-lymphocyte–induced maturation protein-1 (BLIMP-1). IL-21 also decreased IgE production, possibly by an IFNγ-dependent mechanism[38,63,65, 66,69,95,96,97,98,99,100,101]. A recent study suggested that IL-21 can both enhance and inhibit IgE synthesis, depending on B-cell density. In this sense, IL-21 increased IgE production over IL-4/CD40 stimulation at lower cell concentrations and significantly reduced it at higher concentrations[101]. Finally, IL-21 can synergize with BAFF/BLyS in stimulating plasma cell differentiation from a unique population of human splenic memory B cells, contributing to serologic memory in an antigen-independent manner[102].

### NK Cells

NK-cell differentiation and growth is clearly dependent on CD132-dependent cytokines, as demonstrated by CD132-deficient mice that lack mature NK cells[18]. In this sense, IL-21, in combination with IL-15 and fms-related tyrosine kinase 3-ligand (FLT3 ligand), was shown to increase the proliferation and differentiation of $CD34^+$ precursors in mature NK cells[24,103,104]. In addition, IL-21 increased the proliferation and the effector functions of mature NK cells, including IFNγ production by a mechanism independent of NF-κB[24,56,65,103,104,105,106]. It was observed that IL-21 differently affects human NK-cell subsets, in respect to proliferation and cytotoxicity[61]. IL-21 also induced changes in the $CD56^+$ cell cytokine secretion profile, as IL-21 increased levels of IL-10 and granulocyte macrophage colony-stimulating factor (GM-CSF), and decreased TNFα levels[104]. It was further proposed that IL-21 is able to channel NK-cell function by altering the pattern of activation/costimulatory receptors. In this way, IL-21 down-regulates NKG2D/DAP10 and Ly49F expression, while increasing the expression of the NK activation receptors NKp30 and 2B4[85,107]. A property to limit the innate response was also attributed to IL-21, since it is able to limit the activation of NK cells by inhibiting naive cell expansion and increasing apoptosis of mature cells[65,108]. Finally, using an experimental model of human autoimmune myasthenia gravis induced by the acetylcholine receptor self-antigen, Liu et al. showed that NK cells undergo proliferation during the initiation of autoimmunity, followed by degeneration associated with the establishment of the autoreactive T-cell response. The NK-cell degeneration was mediated by IL-21 derived from autoreactive $CD4^+$ T cells. On the contrary, IL-21Rα–deficient mice, with competent NK cells, developed exacerbated autoimmunity. This suggests that IL-21 might be involved in NK-cell dysfunction and death in order to control excessive autoimmunity[109].

#### NKT Cells

A recent study from Coquet et al. evaluated the effect of IL-21 on NKT-cell function. It was shown that IL-21 alone is able to enhance NKT-cell survival *in vitro*, and to increase their proliferation in combination with IL-2 or IL-15. IL-21 also enhanced granular morphology, expression of granzyme B and inhibitory receptors Ly49C/I and CD94, and cytokine production in response to anti-CD3/CD28. Finally, it was suggested that NKT cells may be subject to autocrine IL-21–mediated stimulation, since these cells were demonstrated to be potent producers of IL-21[39].

## Myeloid Cells

### Dendritic Cells

It was demonstrated that *in vitro* differentiation of bone marrow–derived dendritic cells in the presence of IL-21 leads to an immature phenotype and inhibits their activation[26]. In this sense, it was observed that IL-21 inhibits dendritic cell–mediated T-cell activation and induction of contact hypersensitivity *in*

*vivo*[110]. It was suggested that these functionally immature dendritic cells play a role in either inducing anergy or regulating the differentiation of naive T cells toward a suppressor phenotype[96]. Also, IL-21 was shown to inhibit LPS-stimulated dendritic cell maturation and expression of CD86 and HLA class II, in addition to reducing LPS-stimulated production of TNFα, IL-12, CCL5/RANTES, and CXCL10/IP-10. A possible mechanism explaining the effects of IL-21 in dendritic cells is that IL-21 enhances SOCS1 and SOCS3 gene expression, two molecules known to suppress cell functions and to interfere with TLR4 signaling[111]. Beside the possible involvement of SOCS, the inhibitory function of IL-21 on dendritic cell maturation may also be related to Jak3, found to regulate negatively dendritic cell cytokine production and survival[112]. More recently, it was shown that immature myeloid dendritic cells pretreated with IL-21 lead to an increased IFNγ production by NKT cells on stimulation with α-GalCer[113]. Thus, IL-21 seems to be able to trigger dendritic cells to modulate NKT-cell functions instead of activating conventional T cells.

## Neutrophils, Monocytes, and Macrophages

We have recently investigated the potential expression of IL-21Rα by neutrophils, monocytes, and macrophages, and the potential effect of IL-21 on their functions[46]. We demonstrated that IL-21Rα is not expressed in resting neutrophils or following stimulation with classical agonists (fMet-Leu-Phe, LPS, PMA, and IL-15) or by IL-21 itself. This absence of expression correlates with the inability of IL-21 to modulate several important neutrophil responses, including apoptosis, superoxide production, phagocytosis, RNA synthesis, and cytokine/chemokine secretion[114,115,116]. We also demonstrated that IL-21 is not a chemotactic factor for neutrophils. In contrast, promyelocyte HL-60 cells were found to express IL-21Rα and its expression was down-regulated during their differentiation toward the neutrophil phenotype, supporting the fact that mature neutrophils do not express IL-21Rα. In contrast, IL-21Rα was detected in HL-60 cells differentiated toward the monocyte or macrophage phenotype. Concomitant with this, IL-21Rα was found to be expressed in both human monocytes and monocyte-derived macrophages, as detected by western blot studies. Monocyte-derived macrophages, but not monocytes, were found to secrete CXCL8/IL-8 following activation with IL-21[46]. Interestingly, it was recently observed that monocyte-derived macrophages express low levels of IL-21Rα mRNA, but not monocytes[111], suggesting a differential expression of IL-21Rα that could explain the difference observed in IL-21–induced CXCL8/IL-8 secretion. In addition, IL-21R expression was observed in murine macrophages and IL-21 modulated innate and acquired immune functions in these cells[117]. Synovial macrophages were also shown to express IL-21Rα, suggesting a potential role for IL-21 in the regulation of inflammatory response[47]. Pesce et al. recently observed that IL-21 increased CD124 and CD213a (IL-13Rα1) expression in bone-marrow–derived macrophages, resulting in increased FIZZ1/RELMα mRNA and arginase activity-1 following stimulation with IL-4 and IL-13. These results suggest that IL-21Rα (and indirectly IL-21) is an important amplifier of alternative macrophage activation by Th2-type cytokines (IL-4 and IL-13), as opposed to the classical macrophage activation by IFNγ, IL-12, and IL-18[74].

The role of IL-21Rα in immature myeloid cell physiology is currently unknown. We have reported that IL-21 induced ERK1/2 phosphorylation in HL-60 cells, but this was not associated with proliferation or differentiation[46]. More recently, we have demonstrated that IL-21 is unable to induce proliferation and to delay factor deprivation-induced apoptosis of bone marrow cells of myeloid origin ($CD11b^+$); this has been correlated with the absence of IL-21Rα expression in these cells. In contrast, IL-21 inhibited apoptosis of bone marrow cells of lymphoid origin ($CD11b^-$). Because the bone marrow is mainly composed of mature PMNs, we cannot exclude a potential role of IL-21 on immature myeloid cells[118]. Ozaki et al. recently observed that, contrary to immature c-$Kit^+$ Sca-$1^+$ $Lineage^{-/low}$ cells, bone marrow–derived myelomonocytes lack expression of IL-21Rα, suggesting that IL-21 is able to increase the proliferation of progenitor cells, but not of differentiated myelomonocytes. However, overexpression of IL-21 induced expansion of hematopoietic progenitor cells in the spleen, but not in the bone marrow; only a marginal increase was observed in the latter case. In fact, as suggested by this group, IL-21 might contribute to hematopoiesis in a redundant fashion and could be implicated in inducing the differentiation of hematopoietic cells in combination with another cytokine, such as G-CSF, GM-CSF, or IL-7[68].

## ANTITUMORAL EFFECTS OF IL-21: A POTENTIAL IMMUNOTHERAPEUTIC AGENT IN CANCER

The antitumor effects of IL-21 have been observed in many preclinical models of tumor immunotherapy. Among others, promising antitumoral effects of IL-21 were observed in models of bladder and pancreatic cancer, colon carcinoma, fibrosarcoma, and mammary carcinoma, and were mainly related to an increased NK and $CD8^+$ T-cell activity[42,60,108,119,120,121,122,123,124,125,126,127]. Injection of tumors genetically modified to secrete IL-21, alone or in combination with other cytokines, led to tumor regression and subsequent rejection or, at least, to a significant delay in the tumor growth[82,83,128,129,130,131,132]. The IL-21–enhanced tumor rejection in mice challenged with breast, lung, or prostate carcinoma was shown to be dependent on the NKG2D receptor, an activating type II disulfide-linked homodimeric receptor expressed on NK and $CD8^+$ T cells, indicating that IL-21 therapy may work optimally against tumors that elicit a NKG2D-mediated immune response[133]. In addition, an immunotherapy of micrometastases by an IL-21–based cellular vaccine was showed to be strongly potentiated by the depletion of regulatory T cells[134]. Injection with recombinant plasmid containing IL-21 could also be a potential tumor gene therapy, since suppressed tumor growth was observed in IL-21–treated mice[121,124,135]. In fact, a combinatory gene therapy with electrotransfer of midkine promoter-HSV-TK (herpes simplex virus type 1-thymidine kinase) and IL-21 into the tumors was recently reported and was shown to be an efficient gene therapy with improved safety[136]. More importantly, in contrast to IL-2 and IL-15, two CD132-dependent cytokines known for their potential antitumor activity, IL-21 showed a superior ability to stimulate clonal expansion, differentiation, and survival of tumor-specific $CD8^+$ T cells. In addition, IL-21 induces long-term survival in mice whether the cytokine is delivered at early or advanced stages of the disease[122]. Finally, the combined administration of IL-21 with low-dose IL-2 resulted in an increased long-term tumor-free survival frequency in a murine melanoma tumor model, when compared to the use of each cytokine alone. These animals demonstrated tumor-specific protection after rechallenge with melanoma cells and a higher number of circulating tumor antigen-specific $CD8^+$ T cells predominantly partitioned into central memory or effector memory phenotypes[137].

The immunostimulatory effects of IL-21 led to its usage as an immunotherapeutic agent for the treatment of human cancer. A human phase I clinical trial of IL-21 on metastatic renal cell carcinoma or melanoma was designed based on the antitumor activity observed in a murine model of kidney cancer. A tolerable intravenous dose was determined, with mild to moderate toxicities that resolved after treatment. Several dose-dependent changes in immune parameters were observed, including increased serum levels of soluble CD25 (IL-2R$\alpha$) and up-regulation of perforin and granzyme B mRNA in $CD8^+$ T cells. Clinical regressions of renal cell carcinoma were observed after IL-21 treatment, although the duration of regression has yet to be defined. A phase II study in now in progress[138]. It was suggested that a combination of IL-21 with other immunomodulatory agents (such as other cytokines) and/or antiangiogenic agents may help to achieve a higher proportion of remission in renal carcinoma cancer[139]. Potential treatment strategies using IL-21 were recently evaluated in a mathematical model. The model supported clinical use of IL-21 as a potent stimulator of cellular immunity against cancer, and suggested selecting the immunotherapy strategy according to tumor immunogenicity[140].

## IMPLICATION OF IL-21 IN INFLAMMATION AND DISEASES

The recent observations that the $Th_{17}$ cell subpopulation is the highest producer of IL-21 among the different $CD4^+$ subsets[32,33] strongly supports a role of this cytokine in inflammatory processes. In this respect, inflammatory cell recruitment following IL-21 administration was observed in different animal models[46,83,106,119,135,141], while a potential role as an inflammatory cytokine was associated with IL-21 in fish[142,143]. IL-21 has been associated with different inflammatory and autoimmune diseases, such as rheumatoid arthritis, systemic lupus erythematosus, Crohn’s disease, and neurological autoimmune diseases. This section will present the potential implication of IL-21 in these inflammatory diseases.

## Recruitment of Inflammatory Cells in Experimental Inflammatory Models

Using the murine air pouch model, we recently demonstrated that IL-21 is able to induce the recruitment of both neutrophil and monocytic cell populations. The mechanism responsible for the recruitment of these cell populations is currently unknown, but is independent of local production of IL-6, CCL3/MIP-1α, CCL5/RANTES, and CXCL2/MIP-2. However, we believe that IL-21 may be indirectly responsible for attracting neutrophils *in vivo* via the production of chemokines by other cell types, including the recruited monocytic cell population. Based on a panel of neutrophil functions that have been investigated, and the fact that these cells do not express IL-21Rα, we concluded that, although it is not a neutrophil agonist by itself, IL-21 should be considered as a proinflammatory cytokine[46]. Inflammatory infiltrates in multiple tissues, consisting of macrophages and neutrophils, as well as increased numbers of myeloid cells in spleen and bone marrow, were also observed in transgenic mice overexpressing IL-21[141]. Inflammatory infiltrates and central nervous system inflammation was also enhanced when IL-21 was administered before induction of experimental autoimmune encephalomyelitis[106]. Using the hydrodynamics-based gene delivery technique, Wang et al. were able to increase the levels of circulating IL-21 in mice, which resulted in altered splenic and peripheral blood subpopulations. Among the different cell types, the percentage of cells in the myelomonocytic lineage, as defined by CD11b and Gr-1 cells, was increased after murine IL-21 administration[119]. In another study, adenocarcinoma cells genetically modified to secrete IL-21 were injected into mice that developed small tumors with numerous infiltrating granulocytes, NK cells, and $CD8^+$ T cells. *In vivo* depletion experiments by specific antibodies showed that rejection of the tumors required $CD8^+$ T lymphocytes and granulocytes[83]. Finally, injection of a plasmid DNA encoding IL-21, used as an adjuvant to enhance CD8+ T-cell responses and tumor immunity, resulted in increased inflammatory cell infiltrates, identified as a mixture of mononuclear myeloid cells and neutrophils[135]. Altogether, these studies strongly suggest a role for IL-21 in the recruitment of inflammatory cells *in vivo*.

## Bacterial and Viral Infection

Reduced granulomatous inflammation and liver fibrosis were observed in *Schistosoma mansoni*–infected IL-21$R\alpha^{-/-}$ mice and wild-type mice treated with soluble IL-21Rα-Fc. The impaired granulomatous response was associated with reduced Th2 cytokine expression and function, as evidenced by attenuated IL-4, IL-13, acidic chitinase (AMcase), FIZZ1, and Ym1 mRNA expression. A similar impaired Th2 response was observed following *Nippostrongylus brasiliensis* infection. This suggests that, as an important amplifier of alternative macrophage activation, IL-21Rα plays an essential function in the development of pathogen-induced Th2 responses, in addition to its important role in Th2-driven inflammation[74]. Recently, IL-21 was shown to be constitutively expressed in gastric mucosa. A more abundant expression of this cytokine was observed in biopsy specimens and purified mucosal $CD3^+$ T cells from *Helicobacter pylori*–infected patients. Furthermore, it was demonstrated that gastric epithelial cells express IL-21Rα and CD132, and respond to IL-21 by increasing matrix metalloproteinases (MMP)-2 and MMP-9 production through NF-κB activation. Consistently, treatment of *H. pylori*–infected gastric explants with anti-IL-21 reduced epithelial cell–derived MMP-2 and MMP-9 production. Therefore, IL-21 might be involved in mucosal ulceration and epithelial damage in *H. pylori*–infected subjects[144].

A recent study evaluated the ability of a plasmid-derived IL-21, delivered alone or in combination with the IL-15 gene, to regulate immune responses to the HIV-1 envelope (Env) glycoprotein induced by DNA vaccination in a mouse challenge model. It was observed that IL-21 produced sustained resistance to viral transmission when injected 5 days after DNA vaccination. The vaccine-induced recall responses were augmented by the synergistic combination of IL-21 and IL-15, and were related to the expansion of $CD8^+$ $CD127^+$ memory T-cell pools specific for a subdominant Env epitope, enhanced $CD8^+$ T-cell function, increased antibody-dependent cellular cytotoxicity, and complement-dependent lysis of Env-expressing target cells. These results indicated that plasmid-delivered IL-21 and IL-15 can increase the magnitude of the response to DNA vaccines[84]. Moreover, IL-21 was found to up-regulate perforin expression of $CD8^+$ T cells, including memory and effector subsets and virus-specific T cells, from HIV-infected patients. This response was not associated with TCR-induced degranulation and induction of T-cell activation or proliferation, indicating that $CD8^+$ T cells from HIV patients can be modulated by IL-21 to increase perforin expression without undergoing overt cellular activation[145]. The above results indicate that IL-21 could potentially be useful in anti-HIV immunotherapy.

## Rheumatoid Arthritis

Rheumatoid arthritis is a common autoimmune disease resulting in progressive destruction of joints, as well as a variety of extra-articular manifestations, mediated by $CD4^+$ T cells, B cells, macrophages, neutrophils, and synovial fibroblasts. IL-21Rα was shown to be expressed by rheumatoid arthritis synovial macrophages and synovial fibroblasts, and was associated with the activated phenotype of rheumatoid arthritis synovial fibroblasts. However, it negatively correlated with the destruction of articular cartilage and bone. IL-21 was not detectable in synovial biopsy samples from rheumatoid arthritis patients, but the possibility that this cytokine is produced outside the synovium in patients with rheumatoid arthritis cannot be excluded[47]. More recently, immunohistochemical staining demonstrated that IL-21Rα–positive cells were significantly increased in inflamed synovial tissues of rheumatoid arthritis patients compared with osteoarthritis patients and healthy controls. IL-21Rα was mainly expressed in $CD4^+$, $CD8^+$, B cells, and NK cells. Stimulation of peripheral blood and synovial fluid T cells from rheumatoid arthritis patients with an anti-CD3 antiboby and IL-21 enhanced CD69 expression, proliferation, and TNFα and IFNγ cytokine production when compared with controls. This indicates that T cells from rheumatoid patients are more responsive to IL-21, suggesting that the blockade of IL-21R signaling may have a therapeutic potential in rheumatoid arthritis patients[146]. The effect of IL-21 was also examined in murine and rat experimental models of arthritis, in which arthritis was induced with collagen in DBA/1 mice and with Freund's complete adjuvant in Lewis rats. These animals were treated with IL-21R.Fc, a fusion protein containing the extracellular domain of mouse IL-21Rα fused to the constant region of a mutated mouse IgG2a. This fusion protein was shown to neutralize both murine and rat IL-21 bioactivities *in vitro*. DBA/1 mice treated with IL-21R.Fc showed reduced clinical and histologic signs of collagen-induced arthritis when compared with control Ig-treated mice. In response to treatment, the levels of serum IL-6 and nonspecific IgG1 were decreased, while decreased levels of IL-6 mRNA and increased levels of IFNγ mRNA were detected in the paws. Lower levels of IL-6 and IL-17, and higher levels of IFNγ, were observed in collagen-specific spleen cell responses from IL-21R.Fc-treated mice. Thus, blockade of IL-21 in collagen-induced arthritis may function, in part, by modulation of IL-6–driven inflammation and down-regulation of IL-17. Treatment of Lewis rats with IL-21R.Fc resulted in reversal of disease signs and improvements in histologic parameters. These results demonstrate a pathogenic role for IL-21 in animal models of rheumatoid arthritis and suggest that IL-21 may be an interesting therapeutic target for rheumatoid arthritis in humans[147].

## Systemic Lupus Erythematosus

IL-21 has been associated with the development of systemic lupus erythematosus (SLE), an autoimmune disease characterized by dysregulated interactions between autoreactive lymphocytes and the development of antinuclear antibodies. Serum levels of IL-21, IgG1, and IgG3 were significantly elevated in the BXSB-Yaa autoimmune mouse model of SLE, raising the possibility that IL-21 accounts for the hypergammaglobulinemia and class switching to IgG isotypes found in these mice[69]. The decreased expression of IL-21Rα observed on peripheral B lymphocytes in SLE might also account for the pathological features of SLE, such as B lymphocytopenia[148]. Recently, it was shown that the *sanroque* mouse strain failed to repress T-cell functions causing diabetes, and developed high titers of autoantibodies and a pattern of pathology consistent with lupus. The *sanroque* mutation was attributable to the Roquin protein, a RING-type ubiquitin ligase protein family member, and caused the formation of excessive numbers of follicular helper T cells and germinal centers. It was observed that the mutation disrupts Roquin repressor action on ICOS, an essential costimulatory receptor for follicular helper T cells, and results in excessive production of IL-21. These results suggest a role for IL-21 in the excessive autoantibody production[29]. More recently, the impact of blocking IL-21 in the lupus-prone MRL-$Fas^{lpr}$ using an IL-21R.Fc fusion protein was examined. Compared to the control animals, mice treated for 10 weeks with IL-21R.Fc were found to have reduced proteinuria, fewer IgG glomerular deposits, no glomerular basement membrane thickening, reduced levels of circulating dsDNA autoantibodies, and total sera IgG1 and IgG2a. These mice also had reduced skin lesions and lymphadenopathy, a reduced number of splenic T lymphocytes, and altered splenic B-lymphocyte functions *ex vivo*. These data indicate that IL-21 has a pathogenic role in lupus by influencing B-cell function and regulating the production of pathogenic autoantibodies, and suggest that blocking IL-21 in SLE patients may represent a promising novel therapeutic approach[149].

## Systemic Sclerosis

Systemic sclerosis is a connective tissue disorder characterized by vascular abnormalities and excessive collagen synthesis. Collagen accumulation leads to enhanced thickness of vital organs (lungs, kidneys, heart) and ultimately decreases their functions[150]. It is possible that IL-21R signaling could be involved in the pathogenesis of systemic sclerosis via the induction of vascular endothelial growth factor (VEGF). Indeed, IL-21Rα was detected in samples of epidermis from systemic sclerosis patients, whereas no signal was detected in skin specimens from healthy controls. In addition, IL-21Rα was coexpressed with VEGF, a cytokine responsible for the disturbed vessel morphology in the skin of patients with systemic sclerosis[48].

## Crohn’s Disease and Inflammatory Bowel Disease

Crohn’s disease is a chronic inflammatory disorder of the gastrointestinal tract where the excessive production of TNFα, IFNγ, and IL-12 by activated Th1 lymphocytes contributes to the pathogenesis[151]. IL-21 might also contribute to the development of the disease. IL-21 was detected at the inflammation sites of Crohn’s disease patients, while IL-12 enhanced IL-21 expression in normal lamina propria lymphocytes. The blocking of IL-12 in Crohn’s disease lamina propria mononuclear cells caused a decrease of anti-CD3–stimulated IL-21 expression and inhibited IFNγ production. Therefore, it was proposed that IL-21 contributes to the ongoing Th1 mucosal response in Crohn’s disease[62]. In addition, intestinal fibroblasts were shown to express constitutively both IL-21Rα and CD132. IL-21 enhanced production of MMP-1, MMP-2, MMP-3, and MMP-9, but not tissue inhibitors of MMP-1 and MMP-2. MMP synthesis was enhanced with a combination of IL-21 and TNFα. This suggests that blocking IL-21

could help to limit the tissue damage observed in inflammatory bowel diseases[152]. Finally, it was shown that IL-21Rα is constitutively expressed in intestinal epithelial cells, but its expression is higher in inflammatory bowel disease patients. Stimulation of intestinal epithelial cells with IL-21 resulted in increased synthesis of CCL20/MIP-3α, a T-cell chemoattractant. Moreover, a treatment of inflammatory bowel disease explants with anti-IL-21 reduced the production of the CCL20/MIP-3α. These results indicate that IL-21 is involved in the cross-talk between epithelial and immune cells in the gut[64,153].

## Allergy and Asthma

An interesting study showed that administration of IL-21 to mice during immunization with ovalbumin decreased antigen-specific IgE concentrations and the recruitment of eosinophils into the airways of mice that inhaled ovalbumin. Correspondingly, IL-21 also inhibited the induction of germline Cε by LPS and IL-4, which is required for IgE class switching[95]. Because IL-21 can regulate IgE production and eosinophil recruitment, targeting the IL-21/IL-21R system could be of clinical interest for the treatment of asthma and allergies.

## Experimental Autoimmune Encephalomyelitis

Proinflammatory $Th_{17}$ cells are thought to be involved in the induction of experimental autoimmune encephalomyelitis (EAE), an animal model of human multiple sclerosis[154]. Nurieva et al. recently observed an amelioration of the disease when EAE was induced in IL-21–deficient mice, compared to EAE induced in wild-type mice. A further analysis of the $CD4^+$ cells infiltrating the central nervous system showed that these cells express IFNγ, but not IL-17, while $CD4^+$ cells from wild-type highly express IL-17. Therefore, it was suggested that the lack of IL-21 impaired $Th_{17}$ differentation *in vivo* and protected against EAE[33]. Another group also suggested that IL-21 could be involved in the development of EAE by affecting NK cells. It was observed that IL-21 administration before induction of EAE with a neuroantigen and an adjuvant enhanced the inflammatory influx into the central nervous system, the severity of the disease, and NK-cell functions, including IFNγ secretion. In contrast, if IL-21 was administered after EAE progression or if NK cells were depleted, no effects were observed[106]. Finally, since IL-21 was found to regulate the reciprocal development pathway of generation of $Th_{17}$ and $T_{reg}$ cells, it was proposed that the enhanced autoimmunity observed following IL-21 administration could be likely related to increased differentiation of $Th_{17}$ cells and suppression of $T_{reg}$ generation[32]. Therefore, these data strongly suggest a role for IL-21 in the development of some neurological autoimmune diseases.

## Autoimmune Diabetes

Development of autoimmunity in nonobese diabetic (NOD) mice is associated with lymphopenia and elevated expression of IL-21 and IL-21Rα by activated T cells. Since neither overexpression of IL-21 nor lymphopenia alone can induce diabetes in these mice, development of autoimmunity appears to result from the combination of lymphopenia and increased IL-21 production. It has been suggested that T cells expressing IL-21Rα tend to readily undergo cell death, which leads to the observed lymphopenia. In these lymphopenic settings, T cells, in the absence of competition, strongly interact with self-antigen–bearing dendritic cells and robustly respond to growth-promoting cytokines. Therefore, islet-specific T cells in lymphopenic conditions most likely receive a strong TCR signal in the pancreatic draining lymph nodes, survive and acquire effector functions against self-antigen, resulting in the development of the disease[155,156,157,158,159]. The link between IL-21 and autoimmune diabetes was recently reinforced with the observed association of polymorphism of IL-21 and IL-21Rα genes with type 1 diabetes[160].

## CONCLUDING REMARKS

Since the discovery of IL-21Rα and IL-21, a multitude of functions has been attributed to this cytokine. IL-21 plays a predominant role in the regulation and production of immunoglobulin, and in the regulation and activation of B, T, and NK cells. Its effects are not limited to the lymphoid compartment, as it was demonstrated that IL-21 down-regulates the functional maturation of dendritic cells, acts as an amplifier of alternative macrophage activation, and indirectly activates neutrophils. The antitumoral effects of IL-21 clearly makes it an ideal candidate for immunotherapy and, in this sense, the current clinical trials are promising. However, increased expression of this cytokine has also been associated with autoimmune and inflammatory diseases, such as rheumatoid arthritis and Crohn's disease. Knowing that $T_{reg}$ cells play an important role in maintaining self-tolerance, targeting IL-21 in autoimmune and inflammatory diseases will help to restore the balance between pathogenic $Th_{17}$ and $T_{reg}$ cells, the latter cell type being defective in autoimmune diseases. In addition, since IL-21 represents a proinflammatory potential of great significance, its use as an antitumor agent must be carefully examined in order to achieve the desirable effects without triggering autoimmune and inflammatory diseases as a side effect. The further characterization of IL-21 in physiological and pathological conditions will help to determine the correct balance between IL-21–induced antitumoral immunity and IL-21–induced autoimmunity.

## ACKNOWLEDGMENTS

This work was supported by grants to D.G. from the Natural Sciences and Engineering Research Council of Canada and by grants to D.G. (MOP-14416) and a Post-doctoral Research Award to M.P. from the Canadian Institutes of Health Research. D.G. is a Senior Scholar of the Fonds de la Recherche en Santé du Québec. We thank Mary Gregory for reading this manuscript.
